# 

**Published:** 1896-09

**Authors:** 


### BOOKS RECEIVED.

Twentieth Century Practice. An International Encyclopedia of Modern Medical Science. By leading authorities of Europe and America. Edited by Thomas L. Stedman, M. D., New York City. In twenty volumes. Volume VIII. "Diseases of the Digestive Organs." New York : Willam Wood & Company. 1896.

Eighteenth Annual Report of the State Board of Health of the State of Illinois. (Being for the year ended December 31. 1895.) With an Appendix containing the Official Register of Physicians and Midwives, 1896. Springfield, Ill. : Ed. F. Hartman, State Printer. 1896.

The Eye and Its Care. By Frank Allport, M. D., Minneapolis, Minn., Professor of Clinical Ophthalmology and Otology in the Minnesota State University; President of the Minnesota State Medical Society; Secretary of the ophthalmological section of the American Medical Association, etc., etc. Philadelphia: J. B. Lippincott Company. 1896.

The American Academy of Railway Surgeons. Report of the second annual meeting, held at Chicago, Ill., September 25, 26 and 27, 1895. Edited by R. Harvey Reed, M. D., Columbus, O. Chicago: American Medical Association Press. 1896.

A Treatise of Appendicitis. By John B. Deaver, M. D., Surgeon to the German Hospital, Philadelphia. Containing thirty-two full-page plates and other illustrations. Philadelphia: P. Blakiston, Son & Co., 1012 Walnut street. 1896.

A Treatise on Surgery. By American authors. Edited by Roswell Park, M. D., Professor of Surgery and Clinical Surgery, Medical Department, University of Buffalo, Buffalo, N. Y. In two very handsome octavo volumes, comprising about 1,600 pages, with about 800 engravings, largely original, and about forty full-page plates in colors and monochrome. Volume I., General Surgery and Surgical Pathology. Volume II., Special Surgery. Price, per volume, cloth, $4.50; leather, $5.50; net. Lea Brothers & Co. 1896.

